# Recent advances in the treatment of *Clostridioides difficile* infection: the ever-changing guidelines

**DOI:** 10.12703/b/9-13

**Published:** 2020-11-18

**Authors:** Shruti Khurana, Alyssa Kahl, Kevin Yu, Andrew W DuPont

**Affiliations:** 1 Department of Internal Medicine and Pediatrics, The University of Texas Health Science Center at Houston, Houston, TX, USA; 2 Department of Internal Medicine, Baylor College of Medicine, Houston, TX, USA; 3 Department of Internal Medicine, The University of Texas Health Science Center at Houston, Houston, TX, USA; 4 Associate Professor, Department of Gastroenterology, Hepatology and Nutrition, The University of Texas Health Science Center at Houston, Houston, TX, USA

**Keywords:** Clostridium Difficile Infection, IDSA, Fecal Microbiota Transplantation, CDI

## Abstract

*Clostridioides difficile* infection (CDI), formerly known as *Clostridium difficile*, continues to be the most common healthcare-associated infection worldwide. With the shifting epidemiology towards higher a incidence of community-acquired CDI and the continued burden on the healthcare system posed by high rates of CDI recurrence, there has been an impetus to advance the diagnostic testing and treatment strategies. Recent advancements over the past decade have led to rapidly changing guidelines issued by the Infectious Diseases Society of America and European Society of Clinical Microbiology and Infectious Diseases. With our comprehensive review, we aim to summarize the latest advances in diagnosing and treating CDI and thus attempt to help readers guide best practices for patient care. This article also focusses on cost-effectiveness of various therapies currently available on the market and provides an analysis of the current evidence on a relatively new monoclonal antibody therapy, Bezlotoxumab, to treat recurrent CDI.

## Introduction

*Clostridioides difficile* infection (CDI), formerly known as *Clostridium difficile*, is the most common healthcare-associated infection worldwide. In the year 2011, CDI was responsible for almost half a million infections and caused approximately 29,000 deaths in the United States alone^1^. Hospital-acquired CDI (HA-CDI) cases lead to increased length of stay and result in a significant financial burden on the healthcare system^2^. In 2015, the total annual CDI-attributable cost in the United States was estimated to be about 6 billion USD^2^. Outcomes for a patient infected with *C. difficile* range from asymptomatic colonization to severe diarrhea, which can progress to toxic megacolon, bowel perforation, septic shock, and even death. However, the incidence of CDI is decreasing globally. This has been attributed to the rapidly decreasing prevalence of the hypervirulent BI/NAP1/027 strain, which led to major outbreaks across North America during the mid-2000s^1,3–6^. Increased antibiotic stewardship programs in hospitals have also significantly reduced the incidence and colonization of CD during recent years^7^.

With the introduction of more-sensitive *C. difficile* assays such as nucleic acid amplification tests (NAATs), more cases of CDI are being detected; however, not all of these cases result in clinically significant CDI that requires treatment^8^. Community-acquired CDI (CA-CDI) has also gained much attention in the past decade^5,9,10^. It is estimated that almost 40% of all CDI cases are community associated^9^. CA-CDI affects younger populations and those without antibiotic exposures who were traditionally thought to be at lower risk for CDI infections^3,9^. Moreover, in the era of molecular testing, several other toxigenic strains have been identified from animal sources and the environment with varying antibiotic susceptibility in different parts of the world, most notably ribotypes 078 and 244^11–13^. The shifting epidemiology of CDI concerns regarding over-diagnosis and over-treatment, and rising costs of the United States healthcare system have created an impetus for continued research in the detection and treatment of clinically significant CDI.

With the recent changes in the guidelines for the management of CDI, we aim to summarize the latest advances in the field of diagnosis and treatment of CDI and thus help readers guide patient care.

## Risk factors

*C. difficile* causes alteration of the intestinal microbiota that allows spores of *C. difficile* to proliferate in the gut mucosa. Symptomatic infection is thought to be due to the production of toxins A and B that leads to the impairment of epithelial barrier function through disruption of cell–cell tight junctions^14^. Current or recent (within 8 weeks) use of antibiotics is one of the most important risk factors in CDI^1,2,9^. Antibiotics like clindamycin, third-generation cephalosporins, amoxicillin, and fluoroquinolones have been implicated in increasing the risk of developing CDI^15^. Antibiotic use increases the risk by at least 8- to 10-fold in the first month and 3-fold in the subsequent 2 months^9^. Thibault *et al*. demonstrated that the risk of development of CDI is directly proportional to the number of antibiotics used and the duration of antibiotic exposure^16^. In a recent study by Hung *et al*., the incidence of CDI was two times higher after 7–11 days of cephalosporin exposure^17^. Other risk factors that predispose patients to CDI include age ≥65 years, exposure to healthcare facilities including outpatient clinics, hospital admissions, and long-term stays in nursing homes. Recent gastrointestinal surgery, especially colonic resection, immunocompromised states (e.g. malignancy, diabetes mellitus, and HIV), exposure to antineoplastic agents, gastric acid suppressants (e.g. proton pump inhibitors [PPIs]), and co-morbidities like inflammatory bowel disease (IBD) and chronic or end-stage renal disease have also been implicated in CDI^15,17–22^. Patients on PPIs have a 65–75% higher incidence of developing CDI than the normal population, as shown by two large meta-analyses^23,24^. Although an association between PPI use and CDI has been established, it has not been proven that PPI use increases the risk of CDI, as *C. difficile* spores are resistant to gastric acid. It is possible that more colonized patients on PPIs are being tested for CDI because of the well-known side effect of diarrhea.

## Update in diagnosis

With the development of highly sensitive diagnostic testing, many efforts have been made to stratify patients with suspected CDI to avoid overuse of resources and inappropriate treatment of asymptomatic carriers/colonizers. Alasmari *et al*. reported that approximately 15% of patients were colonized with toxigenic *C. difficile* spp.** at the time of admission^25^. Kwon *et al*. showed that careful selection of patients based on clinical presentation of unexplained watery diarrhea (Bristol stool scale 6–7; at least three loose to watery bowel movements in a 24-hour period), associated with abdominal pain or cramping, and objective data can substantially increase the positive predictive value of the tests^26^. The first step should always be a comprehensive historical evaluation to rule out other etiologies of diarrhea, including laxative use in the previous 48 hours of symptom onset, administration of chemotherapeutic agents, enteral feeding, intra-abdominal surgical intervention, and co-morbidities like IBD, irritable bowel syndrome, and other non-infectious causes, etc.^26–28^. According to Dubberke *et al*., recent use of laxatives was noted in one in five patients being tested for CDI in the hospital^27,28^. A highly effective method that can be implemented by laboratories is accepting only diarrheal stool samples and rejecting formed stool specimens.

Imaging of patients with suspected CDI is not recommended owing to poor sensitivity as a diagnostic test, with normal computed tomography (CT) reports in approximately 39% of cases^29^. However, patients with a complicated clinical picture who have findings such as colon wall thickening or “mucosal thumbprinting” on abdominal CT are indicative of CDI with a high positive predictive value for infection and should be considered for treatment^29^. Lower endoscopic studies like flexible sigmoidoscopy are generally not recommended but can be useful in the settings of a coexisting colonic pathology, inconclusive lab tests, colonic ileus where stool sample is not available, or acute worsening symptoms when rapid diagnosis is important to determine the need for urgent surgical intervention^30^. The classic “pseudomembranous colitis” has been reported in about half of CDI cases undergoing endoscopy, thus limiting its use as a test of choice for the confirmation of CDI^30^.

Current diagnostic assays include toxigenic culture (TC), cell culture cytotoxicity neutralization assay (CCCNA), enzyme immune assays (EIAs) for toxin A and toxin B, NAATs, and glutamate dehydrogenase (GDH) tests. Table 1 depicts the estimated relative cost, turn-around time, and sensitivity and specificity of each of these tests.

**Table 1.** Properties of *Clostridioides difficile* diagnostic stool studies.

Assay	Utilization	Cost per specimen^36^	Turnaround time	Sensitivity (%)	Specificity (%)
GDH test	Detects enzymatic product of antigen	$	<4 hours	>90	80
NAAT	Detects genes encoding for toxins	$$$	30 minutes to 2 hours	90–100	90–100
EIA	Detects free toxin	$	1 hour	40–100	90–95
TC	Detects toxigenic spores/organism	$$	48 hours to 7 days	>90–100	>90
CCCNA	Detects free toxin and its effects on cells	$$	24–48 hours	65–85	>90

CCCNA, cell culture cytotoxicity neutralization assay; EIA, enzyme immune assay; GDH, glutamate dehydrogenase; NAAT, nucleic acid amplification test; TC, toxigenic culture.

TC and CCCNA have been considered reference tests for many decades. TC involves inoculation of a stool sample to an anaerobic medium for 2 days up to a week, followed by identification of colonies by Gram stain, colony morphology, and advanced biochemical testing to determine the presence of *C. difficile.* This is labor intensive and cumbersome and has a long incubation period, which reserves its use as a test for laboratory comparison and epidemiological studies instead of routine diagnostic testing. In CCCNA, a stool filtrate is applied over an appropriate cell line to look for toxin cytopathic effect, followed by confirmation with toxin neutralization assay. The disadvantages include variable sensitivity, lack of standardization at testing sites, expertise required for handling and maintenance of stool samples, and subjective variability in the interpretation of results.

There are many commercially available EIA kits that use monoclonal or polyclonal antibodies against toxins A and B produced by *C. difficile.* It is a quick and inexpensive test but comes with several drawbacks, including poor sensitivity, high rates of false positives, and inter-laboratory variations. GDH is an enzyme produced by all strains of *C. difficile*. GDH tests are rapid and economical with a very high sensitivity of over 90%. Since this enzyme is produced by both toxigenic and non-toxigenic strains of *C. difficile*, these tests have a very low specificity and should be used only as a screening assay. This was supported by a recent pre- and post-implementation study performed by Vogelzang *et al*. The authors showed that owing to a high negative predictive value of 98.8%, using GDH as a screening tool followed by testing positive samples with EIA for toxin and later on with NAAT significantly reduced the patient isolation time as opposed to using NAAT alone (28 hours vs. 50.8 hours; *P* <0.001)^31^.

There are inconclusive data regarding the utility of EIA in clinical practice. In 2013, Planche *et al*. reported that EIA A/B-positive cases have worse clinical outcome than TC-positive but stool toxin-negative cases^32^. Around the same time, Humphries *et al*. conducted a study with NAAT and EIA with TC as standard reference to determine the correlation of EIA positivity with disease severity. NAAT was positive in 98% of samples, while 49% and 58% of patients tested EIA positive in mild and severe CDI, respectively. This concluded that there is no significant difference in clinical symptoms and severity in stool toxin-positive cases^33,34^.

NAAT became commercially available in the late 2000s. The commercially available assays detect genes that code for toxins A and B, *tcdA* and *tcdB*, respectively, and binary toxin gene, *cdt*. These assays are fast and are more than 90% sensitive and specific in comparison to TC^35^. Apart from the steep cost difference compared to EIAs and GDH tests and low positive predictive value, there are some concerns with the clinical interpretation of positive tests in asymptomatic colonizers, and a notable increase in the incidence of CDI has been seen since the implementation of NAATs in healthcare facilities^27^.

The concept of combination testing or multistep algorithm was a major highlight of the newly revised guidelines published by the Infectious Diseases Society of America (IDSA) and Society for Healthcare Epidemiology of America (SHEA)^5^. The multistep algorithm comprises combination testing with GDH test plus EIA, NAAT plus EIA, or GDH test plus EIA arbitrated by NAAT in pre-defined criteria for testing stool samples. This two-step algorithm was also supported by the European Society of Clinical Microbiology and Infectious Diseases (ESCMID) and proposed using either GDH test or NAAT for screening followed by reflex testing of positive samples with EIA to confirm the *Clostridioides* present is toxigenic^37^. It has been shown by multiple authors that stool samples that are found to be positive by NAAT followed by a multi-step toxin test have a higher chance of detecting cases of clinically significant disease and are associated with a higher risk of developing CDI-related complications (megacolon, need for colectomy, admission to intensive care unit, 30-day all-cause mortality, duration of diarrhea, readmission, and recurrence) as opposed to only NAAT positive cases^38,39^.

Ignatius *et al*. performed a study to assess the validity of ESCMID guidelines in testing patients for CDI in the outpatient setting. Of the 9,802 stool samples studied, approximately 90% concordance was noted between GDH and EIA testing (95% confidence interval [CI] 89.2–90.4%). However, of the discordant GDH+/EIA– samples, 68% were positive by NAAT (95% CI 64.7–71.0%), and of the discordant GDH–/EIA+ samples, 85% were negative by NAAT (95% CI 71.2–93.5%)^40^. It is generally accepted that in facilities that use screening practices (e.g. rule out laxative use), the NAAT can be used as a stand-alone test without confirmatory EIA for toxin.

Fecal biomarkers like fecal calprotectin and lactoferrin have been assessed to determine a correlation of severity of CDI and recurrence risk^41^. These are non-specific markers of inflammation with no concrete data for their utility in the diagnosis or management of CDI^42–44^.

Repeat testing within 7 days of initial test (regardless of the result) or as test-of-cure are not recommended because of a high risk of false-positive results, very low diagnostic yield, and chance of unnecessary prolonged treatment, as more than 60% of patients may have positive results even after treatment because of asymptomatic spore shedding for up to 6 weeks^45,46^.

## Classification

There are several classifications of CDI based on disease severity and epidemiology. The IDSA/SHEA classifies CDI into healthcare-associated (onset of disease on or after the fourth day of admission to a healthcare facility), community-onset healthcare-associated (onset of disease within 4 weeks of discharge from the healthcare facility), and community-associated (sporadic CDI or onset at least 4 weeks after hospital discharge) disease^5^. This sheds light on the continued risk of CDI in patients after discharge due to suppressed immunity and prolonged antibiotic course post-hospitalization.

Despite this proposed classification, the management of CDI is most commonly described based on severity and number of episodes. There are many factors that have been studied to predict treatment response or failure, risk of recurrence, and degree of severity. There is no criterion that has been validated to assess the severity of CDI at presentation; however, the presence of fever (>38.5°C), white blood cell (WBC) count >15 × 10^9^/L, and creatinine >1.5 mg/dL have been associated with severe and complicated CDI^5^.

## Updates in treatment

The goal of treatment is the resolution of diarrhea and prevention of recurrence, thus decreasing the disease burden. Minimizing unnecessary antibiotic exposure and prompt discontinuation of inciting antibiotic agent(s), if possible, play a pivotal role in the management of CDI. Antibiotic stewardship programs and infection control measures (e.g. hand hygiene) are proven to be the most cost-effective methods in significantly reducing the incidence of CDI and its recurrence^7^. Supportive measures like rehydration and correction of electrolyte imbalances should be addressed in all patients diagnosed with CDI.

Empiric treatment is considered inappropriate in suspected CDI cases, with the exception of fulminant CDI and in cases where substantial delay is expected in retrieving diagnostic studies. The rationale is to limit the overuse of antibiotics and prevent the overgrowth of multidrug-resistant pathogens^47^. Empiric therapy also increases the risk of false-negative PCR results on pre-treated stool samples. Sunkesula *et al*. demonstrated that a positive PCR for CDI is converted to negative after 1, 2, and 3 days of treatment^48^. Anti-motility agents like loperamide are generally contra-indicated because of an increased risk of colonic dilation, perforation, and higher mortality^49^.

### Initial CDI

Initial CDI is defined as onset of symptoms with positive diagnostic test and no history of CDI within the previous 8 weeks. Metronidazole had long been the recommended initial treatment of CDI. However, the most recent IDSA/SHEA guidelines published in 2018 replaced metronidazole with oral vancomycin (125 mg given by mouth four times a day for 10 days) or fidaxomicin (200 mg given by mouth twice daily for 10 days) as the first line of treatment^5^. This was a major revelation supported by strong clinical evidence. There are many reasons for metronidazole being replaced as the first line of treatment for initial CDI, including inferiority of metronidazole to vancomycin in achieving clinical cure rates, higher recurrence rates in the first 30 days post-treatment, delayed response to treatment, need for longer antibiotic course of up to 14 days, and concern for neurotoxicity with repeated and prolonged use^50–53^. Despite the higher cost of vancomycin and fidaxomicin compared to metronidazole, the overall cost of repeated hospitalizations and treatment is expected to be lower with initial treatment with vancomycin or fidaxomicin^54,55^.

Fidaxomicin gained impetus as the standard of care for initial episodes of CDI after two randomized, double-blind phase III trials demonstrated non-inferiority of fidaxomicin to vancomycin, with sustained clinical cure rates ranging from 88 to 92% and lower recurrence rates^56,57^. Lower recurrence rates may partly be the result of the narrower activity and specificity for *Clostridioides* of fidaxomicin compared to vancomycin.

Other alternative therapies currently being evaluated include nitazoxanide, fusidic acid, rifaximin, rifampin, bacitracin, tigecycline, teicoplanin, cadazolid, surotomycin, ridinilazole, LFF571, ramoplanin, CRS3123, auranofin, NVB302, thuricin CD, lacticin 3147, and acyldepsipeptide antimicrobials^58–67^. With most drugs in their initial phases of trials, there is a lack of strong clinical evidence of superiority for any of the above antimicrobials when compared to vancomycin and fidaxomicin.

### Recurrent CDI

One of the major hurdles in the management of CDI is recurrence, which is defined as repeat onset of symptoms with positive diagnostic testing within 2–8 weeks of first CDI episode. It is estimated that approximately a quarter of patients will suffer at least one additional episode, especially when treated with metronidazole or vancomycin^68^. This population is also shown to suffer from 33% higher mortality compared to those with only one episode of CDI^69^. The subsequent CDI can be from the previously treated strain (relapse) or from a new strain due to persistent risk factors (re-infection). Whole genome sequencing showed that 75–85% of recurrences are associated with same-strain relapses^70^. Regardless of the strain, the treatment is similar for both causes. Major risk factors associated with recurrence include age ≥65 years, continued use of antibiotics, severe initial bout of CDI, and possibly gastric acid suppression (Table 2)^71,72^.

**Table 2.** List of risk factors for recurrent *Clostridioides difficile* infection.

• Age ≥65 years • Immunocompromised states (diabetes mellitus, HIV) • Gastric acid suppressants (PPIs and H2 blockers) • Hypervirulent strains of *C. difficile* (ribotype 027, 078, or 244) • Inflammatory bowel disease • Recent gastrointestinal surgery (partial colectomy) • Previous *C. difficile* infection(s) • Chronic/end-stage renal disease • Antibiotic use – current or within the past 3 months

HIV, human immunodeficiency virus; PPIs, proton pump inhibitors

The treatment of first recurrence should be by either of the following two regimens: vancomycin in tapered and pulse doses (125 mg four times per day for the first 10 days, followed by 125 mg two times per day for a week, 125 mg once per day for a week, and then 125 mg every 2 or 3 days for 2–8 weeks) or fidaxomicin if vancomycin was used initially. Several studies have shown that pulse and tapered vancomycin has cure rates close to 74% in recurrent CDI (rCDI), especially if standard 10-day vancomycin course or metronidazole was used for the treatment of the previous CDI^73^. The hypothesis of prolonged vancomycin therapy is to eradicate the vegetative spores that were dormant during the 10-day treatment course. On the other hand, the use of fidaxomicin for the initial CDI episode has been associated with lower initial recurrence rate but has not been shown to decrease subsequent recurrences^74^. In a phase III randomized controlled trial performed by Cornely *et al*. to compare fidaxomicin and vancomycin for rCDI (first episode), similar cure rates were noted for both drugs (>90%), but there was a significantly lower rate of second recurrence of CDI in the fidaxomicin arm (35.5% vs. 19.7%, *P* = 0.045)^75^. Despite common clinical practice, continued prophylactic treatment with agents like rifaximin has not been proven to be effective in preventing recurrence in high-risk groups^76^.

### Role of fecal microbiota transplantation

Fecal microbiota transplantation (FMT) is a rapidly emerging therapy that has gained popularity in the past few decades with promising active research. The fundamental concept behind FMT is the delivery of a more physiologic fecal microbiome from a healthy stool donor into the gut of a patient to correct the underlying severe intestinal dysbiosis. Since its first description dating back to the 1980s, multiple systematic reviews and randomized controlled trials have demonstrated the efficacy and safety of FMT for patients who have rCDI. In these patients, FMT was shown to be superior to standard antibiotic therapy with higher clinical cure rates and lower recurrence rates^77–79^. Furthermore, FMT administered after a course of vancomycin is superior to vancomycin alone for rCDI^78,79^. As per the current IDSA/SHEA guidelines, FMT is recommended for patients with multiple recurrences of CDI who have failed appropriate antibiotic treatments^5^.

Several routes of stool delivery have been explored, including delivery via feeding tube, infusion by colonoscopy, enemas, and oral capsules with lyophilized stool. All methods have been shown to have excellent safety profiles and similar cure rates between 82 and 95% after one or two transplants in most studies^80^. Most clinicians, depending on institutional policies, give a brief antibiotic course to FMT recipients prior to the procedure with the intention of decreasing the disease burden.

FMT is well tolerated by patients and has minimal short-term direct adverse effects. Most of the adverse events are related to complications secondary to the procedures for the instillation of the stool including colonic microperforation, gastrointestinal bleeding, and peritonitis. Adverse events related to FMT itself are usually minor, are self-limited, and usually resolve within a few hours. These include abdominal pain, bloating, diarrhea, constipation, and fever^81^.

Phase III clinical trials are currently underway to further investigate the efficacy and feasibility of using FMT as a primary therapy for CDI. A small initial proof-of-concept trial out of Norway showed potential superiority of FMT over traditional antibiotic therapies^82^. Recurrent CDI is a major concern in IBD patients. Unfortunately, the success of FMT has been variable in this group along with the risk of worsening flares, as reported by some authors^83–85^.

One of the challenges with FMT is the lack of standardized protocols for stool screening and selection, as well as standardized donor sources. Careful selection of a healthy donor is a critical step, and strict exclusionary criteria should be applied by centers to prevent iatrogenic transmission of infectious diseases, which now include COVID-19^81^. In 2019, DeFilipp *et al*. reported one such notable case series of two patients acquiring extended-spectrum beta-lactamase *Escherichia coli* bacteremia post-FMT, resulting in the death of one of these patients^86^.

One small observational study of 32 patients looked at establishing stool banks by reconstituting stool samples from single donors with clinical cure rates on par with other FMT studies using heterogenous stool donations^87^. Other studies have explored the logistical barriers as well. The potential use of freeze-thawed stool samples versus fresh feces has shown non-inferiority between the two types of preparations that could potentially lead to the establishment of stool banking for storage and later use^88,89^.

The potential long-term effects of the alteration of a host’s gut microbiota through FMT are unknown. Given the diverse nature of stool donor sources, there may be species of bacteria that are currently of unknown significance and unculturable bacteria that could result in unforeseen health consequences in the recipient. In a 2013 study of 77 patients by Brandt *et al*., in which the investigators followed the recipients of FMT over the course of 3 months, four of the 77 patients (5%) had developed autoimmune or rheumatologic disorders^90^. It is unknown at this time whether the new autoimmune disorders were related to FMT. Further work needs to be conducted to determine the long-term safety profile of this treatment.

### Severe CDI

Severe CDI can be differentiated from less-severe disease by the proposed criteria described before: presence of fever (>38.5°C), WBC count >15 × 10^9^/L, and creatinine >1.5 mg/dL. Initial treatment of severe CDI includes vancomycin 125 mg orally four times daily for 10 days or fidaxomicin 200 mg orally twice daily for 10 days^5^. Patients with severe disease may also benefit from intravenous metronidazole, especially if there is delayed passage of oral antibiotics, as metronidazole is metabolized by the liver and excreted through the biliary system into the small intestine^91,92^. Mucosal disruption in severe disease can cause systemic absorption of vancomycin, and serum vancomycin levels should be monitored in patients with renal failure^93^. FMT has been proposed as first-line therapy for severe cases of CDI. A retrospective cohort study of 111 patients by Hocquart *et al*. found significant improvement in survival in severe CDI (odds ratio 0.08; *P* = 0.001) and suggests the need for further studies to support FMT as a first-line treatment^94^.

### Fulminant CDI

Fulminant CDI is defined as an infection that is complicated by hypotension, shock, ileus, or megacolon. Early diagnosis and treatment are essential, as the disease can rapidly progress and has a high mortality rate close to 44%^95^. Antibiotic therapy for fulminant CDI consists of enteric vancomycin 500 mg (orally or via nasogastric tube) four times daily and parenteral metronidazole 500 mg every 8 hours. In cases of ileus, rectal vancomycin may be administered via enema with caution for risk of colonic perforation (500 mg in 100 mL normal saline per rectum, retained for as long as possible and administered every 6 hours)^5^. Early surgical consultation is warranted for patients who show no improvement with medical therapy or have a rising serum lactate level (≥2.2 mmol/L) or rising WBC count (≥20,000)^96^. Surgical intervention can be life-saving for select patients^96–99^. A systematic review of 1,433 patients undergoing emergency surgery for CDI found the strongest predictors of postoperative mortality to be preoperative intubation, acute renal failure, multiple organ failure, and shock requiring vasopressors^100^. Total colectomy with end ileostomy was associated with the lowest rates of mortality and reoperation; however, less-extensive procedures can be considered for patients with earlier stage disease^100^.

## Role of bezlotoxumab

Bezlotoxumab is a fully humanized monoclonal antibody that neutralizes *C. difficile* toxin B. Two multicenter, phase III, placebo-controlled clinical trials (MODIFY I/MODIFY II) showed patients receiving bezlotoxumab had a significant reduction in the rate of rCDI after 12 weeks of infusion (17% vs. 28% in MODIFY I, *P* <0.001, and 16% vs. 26% in MODIFY II, *P* <0.001)^101^. Although the majority of the patients (94%) received bezlotoxumab within 6 days of initiation of standard-of-care (SOC) antibiotics, there was no difference in rCDI from the timing of bezlotoxumab infusion^102^. In October 2016, the US Food and Drug Administration approved the use of bezlotoxumab as adjunctive therapy combined with standard treatment for CDI in patients with high risk of recurrence. This is administered intravenously during standard CDI treatment as a single infusion of 10 mg/kg over 60 minutes with no need for dose adjustments for renal or hepatic impairment. Though this treatment is well tolerated, infusion-related reactions (generally mild) were noted in 10% of patients. Serious drug-related adverse events (0.5%) were related to infections and cardiac disorders in patients with known congestive heart failure. This led to caution on the use of bezlotoxumab in patients with heart failure^103^. A post-hoc analysis proved that bezlotoxumab reduced rCDI, decreased the incidence of future FMTs, and promoted reduction in 30-day readmissions in patients with one or more risk factors for recurrence^104^. In an analysis looking at inpatient hospital stays for CDI, it was found that patients who were treated with bezlotoxumab had a decreased length of hospitalization^105^. A recent multicenter cohort study performed by Hengel *et al*. tested the efficacy of bezlotoxumab on 200 patients receiving SOC antibiotics. The authors noted a 90-day rCDI prevention rate of 84.1%. The high success rate was noted with all SOC antibiotics and was independent of the time of diagnosis and the time of infusion^106^. In conclusion, bezlotoxumab is a very promising drug, and more head-to-head trials are needed to compare FMT and SOC with bezlotoxumab with regard to cost, efficacy, and safety.

## Cost-effectiveness of various therapies

The management cost of CDI places a large burden on the healthcare system. Recurrent episodes of CDI make patients 12.5 times more likely to accrue inpatient hospital costs due to readmissions and longer hospital stays than patients without recurrence^107^. Cost-effectiveness of therapy is a complex analysis which entails many direct and indirect aspects of resource utilization, including cost of diagnostic testing and medications, cost of hospitalization(s) including ICU care, and cost of contact isolation, readmissions, total length of stay, and management of complications and adverse events^108^.

There are three strategies to mitigate the total costs related to CDI: prevention of initial episode (infection control measures and antibiotic stewardship)^109^, cost-effective treatment of episode(s) with good cure rates, and prevention of recurrence(s). Among the available antimicrobials, metronidazole is the cheapest medication which is readily available in the resource-limited setting, but this is also the least-effective therapy which provided no gain in quality adjusted life years (QALY)^110^ and is no longer recommended as a first-line therapy when fidaxomicin or vancomycin is available. Despite the highest initial cost of fidaxomicin, some studies have found fidaxomicin to be more cost-effective than other treatment^111^; however, in a recent meta-analysis performed by Le *et al*. comparing treatment strategies for initial and rCDI, there were divergent results on the cost-effectiveness of fidaxomicin for initial and rCDI^112^. The findings are particularly conflicting for initial CDI of varying severity (mild–moderate and severe)^113,114^. On the other hand, FMT delivered by colonoscopy has been consistently shown to be the most cost-effective therapy for treating rCDI^112,115–117^.

## The role of probiotics in CDI

There is extensive ongoing research to learn the potential role of probiotics in regulating gut dysbiosis, which in turn predisposes to the development of CDI. Traditionally, there has been limited evidence to support the effectiveness of probiotics in the prevention and treatment of CDI. In a recent meta-analysis, Shen *et al*. evaluated 19 randomized controlled trials that included 6,261 antibiotic-treated hospitalized patients who received *Saccharomyces boulardii*, *Lactobacillus* spp., *Bifidobacterium* spp., and *Streptococcus* spp., alone or in combination, with the aim of preventing the development of CDI. The study showed promising results, with a >50% reduction in the rate of CDI in patients who received concurrent probiotics^118^. Future research will need to focus on elucidating the most-effective probiotic(s), optimum amount, and ideal duration for the prevention of CDI.

## Conclusion

Antibiotic restriction is a vital measure to control CDI. All healthcare facilities should implement an antimicrobial stewardship program, which includes minimizing the frequency, duration, and number of antibiotic(s) whenever feasible. Vancomycin and fidaxomicin are both cost-effective treatments for an initial episode of CDI and preventing recurrence. FMT should strongly be considered in the treatment of rCDI after a second recurrence (third episode). The role of bezlotoxumab and other newer therapies is evolving and needs to be further studied.

## Abbreviations

CA-CDI, community-acquired *Clostridioides difficile* infection; CCCNA, cell culture cytotoxicity neutralization assay; CDI, *Clostridioides difficile* infection; CI, confidence interval; CT, computed tomography; EIA, enzyme immune assay; ESCMID, European Society of Clinical Microbiology and Infectious Diseases; FMT, fecal microbiota transplantation; GDH, glutamate dehydrogenase; HA-CDI, hospital-acquired *Clostridioides difficile* infection; IBD, inflammatory bowel disease; IDSA, Infectious Diseases Society of America; NAAT, nucleic acid amplification test; rCDI, recurrent *Clostridioides difficile* infection; SHEA, Society for Healthcare Epidemiology of America; SOC, standard of care; TC, toxigenic culture; WBC, white blood cell
